# The Perfusion Features of Recurrent Hepatocellular Carcinoma After Radiofrequency Ablation Using Contrast-Enhanced Ultrasound and Pathological Stemness Evaluation: Compared to Initial Tumors

**DOI:** 10.3389/fonc.2020.01464

**Published:** 2020-08-26

**Authors:** Jin-Yu Wu, Xiu-Mei Bai, Hong Wang, Qian Xu, Song Wang, Wei Wu, Kun Yan, Wei Yang

**Affiliations:** ^1^Department of Ultrasound, the First Hospital of Harbin, Harbin, China; ^2^Key Laboratory of Carcinogenesis and Translational Research (Ministry of Education/Beijing), Department of Ultrasound, Peking University Cancer Hospital and Institute, Beijing, China

**Keywords:** hepatocellular carcinoma, radiofrequency ablation, recurrence, contrast-enhanced ultrasound, cancer stem cell

## Abstract

**Objective:** To investigate the perfusion features of local recurrence in hepatocellular carcinoma (HCC) after radiofrequency ablation (RFA) with contrast-enhanced ultrasound (CEUS) and pathological correlation, as well as to compare with those of initial HCC.

**Methods:** From 2010 to 2018, 42 patients with recurrent HCC after RFA were enrolled in this study. The initial HCC patients included 32 males and 10 females with an average age of 58.2 ± 8.1 years. The CEUS images for initial HCC lesions and local recurrence after RFA were compared. The perfusion features were analyzed, including enhancement time, process, boundary, morphology, washout time, washout degree, feeding vessels, and internal necrosis. H&E staining and CD133/EpCAM staining were performed with biopsy samples for the stemness study.

**Results:** According to CEUS, 59.5% of initial HCC lesions had centripetal enhancement, and 61.9% of recurrent HCC lesions had homogeneous enhancement in the arterial phase (*p* < 0.001). A total of 73.8% of initial HCC lesions had well-defined margins at the peak, and 81.0% of recurrent HCC lesions had poorly defined margins (*p* < 0.001). A total of 78.6% of initial HCC lesions had regular morphology at the peak, and 83.3% of recurrent HCC lesions were irregular (*p* < 0.001). Feeding vessels were more frequently found in initial HCC lesion (71.4%) than in recurrent HCCs (38.1%, *p* = 0.002). In the late phase, 60% of initial HCCs had marked washout while 83.3% of recurrent HCC lesion had marked washout (*p* = 0.019). A total of 31.3% of the initial HCC lesions had internal necrosis areas while only 7.1% of recurrent HCC lesions had internal necrosis areas (*p* = 0.035). In tumors 3–5 cm in size, the washout time of recurrent HCCs was shorter than that of initial HCCs (50.3 ± 13.5 s vs. 75.6 ± 45.8 s, *p* = 0.013). Pathological staining showed that the tumor stem cell markers (CD133 and EpCAM) were both highly expressed in recurrent samples compared with initial tumor samples (CD133+: 19 vs. 5%, *p* = 0.002; EpCAM+:15 vs. 6%, *p* = 0.005).

**Conclusions:** Recurrent HCC after RFA had more homogeneous enhancement with a poorly defined border, marked washout, and fewer less feeding vessels and inner necrosis areas compared to initial HCC. The stemness study also found upregulated stemness in recurrent HCC. These specific features might be related to the aggressive biological behavior of recurrent HCC.

## Introduction

In recent years, radiofrequency ablation (RFA) has been widely applied to cure cancers, including liver, kidney, and lung tumors. RFA has been performed as a first-line treatment for hepatocellular carcinoma (HCC) and patients' 5-year survival rates have reached 67.9% (1). Compared to surgical resection, however, RFA has higher local recurrence rates (2, 3), and the second RFA treatment becomes more difficult in the case of large recurrent lesions or recurrent lesions in high-risk locations. Therefore, the accurate evaluation of local recurrence in HCC is an important issue for achieving early diagnosis and conducting timely repeat treatment. Currently, contrast-enhanced computed tomography (CT) and magnetic resonance imaging (MRI) are considered the standard modalities for evaluating RFA efficacy (4, 5). However, CT enhancement involves radioactive radiation, and it is not feasible to use MRI repeatedly in a short period. Contrast-enhanced ultrasound (CEUS) imaging is recognized as the revolution of traditional ultrasound examination and can overcome several limitations of conventional grayscale and color ultrasound techniques (6). Unlike enhanced CT and MRI, CEUS is able to demonstrate dynamic changes in hepatic blood flow in real time (6–10). Furthermore, CEUS provides visible information on the vascular density and structures of lesions and liver tissue (11).

Studies assessing the histopathological features of primary HCC with the perfusion pattern of CEUS are increasing (12–14). Fan et al. (12) showed that ~98% of primary HCC had enhancement in the arterial phase and portal phase with CEUS findings. Wilson et al. (15) reported that washout at parenchymal phase or late phase was another important diagnostic clue for HCC. Based on clinical diagnostic criteria, primary HCC can be diagnosed when the lesion has fast in and fast out features in enhanced images. However, reports on the perfusion features of CEUS in terms of the local recurrence of HCC after local ablation are rare. Recent studies have demonstrated that incomplete RFA of HCC can initiate malignant transition (16, 17). It is not clear whether local recurrent HCC after RFA has specific perfusion characteristics and pathological changes compared to initial tumors. Thus, this study aimed to investigate the difference in CEUS characteristics and pathological features between local recurrent HCC after RFA treatment and initial HCC and to summarize the specific features of recurrent HCC. The results might provide more information about biological behavior after local recurrence and advice for appropriate treatment.

## Materials and Methods

### Patients

From 2010 to 2018, 1,118 consecutive HCC patients received ultrasound-guided percutaneous RFA treatment in our center. The inclusion criteria of liver tumors for ultrasound-guided percutaneous RFA (local curative purpose) were as follows: (1) a tumor size of no more than 5 cm and a tumor number of no more than 3; (2) no direct tumor invasion of adjacent organs or tumor thrombi in the main or lobar portal system; (3) a tumor not invading a main bile duct or being obviously exophytic; (4) a tumor accessible via a percutaneous approach; (5) an international standard ratio <1.6 and a platelet count > 50,000/μl; and (6) no extrahepatic metastasis or local extrahepatic metastasis with good control before RFA.

After RFA treatment, the patients received close follow-up. During the follow-up period, 66 patients were diagnosed with local recurrence after RFA. The inclusion criteria for this study were as follows: (1) the patients had local recurrent HCC after RFA in our center; (2) the patients received CEUS examinations for both initial HCC and recurrent HCC tumors; and (3) the initial tumor or recurrent tumor was not treated by previous therapies such as TACE, radiotherapy, or PEI in 1 month before CEUS examination. Regularly, the HCC patients underwent enhanced CT/MRI in the first month after RFA. The enhanced area around or within the ablation zone was defined as residual tumor at the initial evaluation. Then, the patients with residual tumors would receive the second RFA or other therapies. Furthermore, the patients who had completely sufficient ablation continued the follow-up protocol. The enhanced area around or within the ablation zone during follow-up was defined as local recurrence. Finally, 42 patients who met the inclusion criteria were enrolled in this study: 32 males and 10 females with an average age of 58.2 ± 8.1 years old (range: 35–80 years old). Initial HCC and local recurrent lesions were diagnosed by biopsy (which included 17 initial HCC cases and 13 recurrent HCC cases) and clinical diagnosis (nodule size ≥1 cm, two kinds of imaging modalities with a typical enhanced performance, which included 25 initial HCC cases and 29 recurrent HCC cases). Among them, 11 patients underwent biopsy for both initial HCC and recurrent HCC.

### Ultrasound Equipment

Low mechanical index (MI) and real-time contrast-enhanced harmonic ultrasound examination were performed in this study. The contrast-enhanced agent was SonoVue (Bracco SpA, Italy) suspension, which contained 6.07 mg/ml sulfur hexafluoride (SF6) stabilized by a phospholipid shell (microbubble concentration: 5 mg/ml). The mean diameter of the microbubbles was 2.5 μm. The SonoVue suspension was administered through the cubital vein by bolus injection (2 ml in 1–3 s). The GE LOGIQ E9 ultrasound system (General Electric, Milwaukee, WI, USA) was used for this study. The probe frequency ranged from 2.5 to 6.0 MHz. The MI used in CEUS scanning ranged from 0.11 to 0.14.

### CEUS Examination Method

First, the liver was scanned with conventional grayscale ultrasound to identify the lesions' number and location. The size, morphology, border, echogenicity, echotexture, and flow features of the lesions were observed. Then, the examination was switched to a low-MI harmonic pulse-inversion CEUS mode. After injection of the contrast agent, the lesions were scanned continually for a duration of 5–6 min. The enhancement pattern and washout degree of the lesions were noted and recorded. After acquiring the vascular perfusion information for the target lesion, the whole liver was scanned quickly to detect abnormal washout lesions. The abnormal washout lesions required repeat injection of contrast agent if necessary. CEUS dynamic clips and single-frame static images were stored in an ultrasonic instrument hard disk for further analysis.

### Imaging Analysis

All CEUS images and clips were reviewed retrospectively, and the perfusion patterns of CEUS were analyzed by two radiologists with more than 10 years of experience in liver CEUS (W. J. Y. and Y. W.). The blinded radiologists read the CEUS imaging results, without the information of the pathologic and clinical materials. The two radiologists would discuss and make an agreement in each case. The perfusion features between the two groups were compared.

Based on our definition, the arterial phase of CEUS was started with enhancement in the hepatic artery. The peak occurred when the lesion reached its highest echogenicity. The late phase referred to the whole liver parenchyma, reached the highest level of enhancement and was gradually washed out. The CEUS examination time for one injection was 5–6 min. The CEUS features of the liver lesions included enhancement in the arterial phase, the presence of a feeding artery, lesion margins and morphology at the peak of enhancement, the washout degree in the late phase, and the presence of necrosis areas inside the lesion.

Centripetal enhancement was defined as the first enhancement of the lesion in the periphery and then a gradual filling toward the center. Centrifugal enhancement was defined as the first enhancement of the lesion in the center, gradually reaching the periphery. Homogeneous enhancement referred to the rapid enhancement of the entire lesion and showed a significant increase in echogenicity compared to the same level of liver tissue. A well-defined margin was defined as a distinct difference between the lesion and the surrounding liver. Marked washout was defined as a lesion with almost no enhancement or mostly washed out contrast agent within 2 min after contrast injection (18). A feeding vessel was identified in the arterial phase when contrast flowed directly through the vessel from the hepatic hilum to the lesion. Internal necrosis was considered if no contrast agent entered the area from the arterial to the late phase. Regular lesions had a round shape with well-defined margins.

#### Phase of Enhancement

The enhancement pattern should be described separately for the different phases, which for the liver comprise the arterial, portal venous, and the late phases. “Wash in” used for both qualitative and quantitative analyses, referred to the period of progressive enhancement within a region of interest from the arrival of microbubbles in the field of view to “peak enhancement,” which referred to the arterial phase in this study. The “washout phase” referred to the period of reduction in enhancement that followed peak enhancement, which referred to portal phase to the late phase (19).

### Time–Intensity Curve Analysis

Time–intensity curve (TIC) analysis was performed as a quantitative tool to analyze the difference between the initial HCC and recurrent HCC lesions. The stored DICOM data were evaluated using the built-in TIC analysis software of the LOGIQ E9 system (GE, USA). A region of interest (ROI), mainly including the liver lesion, was selected to obtain the TIC. The ROI sampling frame was chosen in lesions around the central and liver tissues as same a depth as possible to avoid the great vessels and tumor necrosis area. The enhanced time was calculated from the time of contrast agent injection to the contrast agents arrived lesions. The peak time was considered from the contrast agents injected to the contrast agent reaching the maximum level. The washout time referred to lesions with echo intensities lower than the time needed for the liver parenchyma.

### Pathological Evaluation

Eleven patients underwent biopsy for initial HCC before RFA and for recurrent HCC after RFA. Pathological specimens were fixed in formalin and were routinely processed and embedded in paraffin. All tumor slices were subjected to hematoxylin and eosin (H&E) staining for gross pathologic examination. CD133 and EpCAM are regarded as important surface markers of cancer stem cells in HCC (20). CD133 (allophycocyanin, Miltenyi Biotec) and EpCAM (fluorescein isothiocyanate, Stem cell Technologies) immunofluorescent (IF) staining were performed for tumor stemness evaluation. The stained slices were reviewed by an experienced pathologist. Slides were imaged and analyzed by using a microscope (Olympus BX41, Olympus, Japan) and imaging software (Micron; Westover Scientific). The temporal evolution of cellular morphology and the spatial distribution of protein expression were determined first. Quantitative analysis was performed by accounting for the percentage of positively stained cells per high-powered field within the tumor zone. Five random high-powered fields were analyzed for a minimum of five specimens for each marker and were scored in a blinded fashion to remove observer bias. The percentage of CD133/EpCAM-positive cells was evaluated with pathological initial HCC and recurrent HCC samples and was compared to observe the changes after RFA treatment.

### Statistical Analysis

All data are expressed as the mean ± standard deviation. The significance of differences in the baseline characteristics, enhancement patterns, ITC parameters, and pathological results were compared by the chi-squared test or independent-sample *t*-test. Statistical significance was defined as a *p*-value < 0.05. All data analysis was performed by using SPSS statistical software 24.0 (SPSS, Chicago, IL, USA).

## Results

### General Features

The enrolled HCC patients included 32 males and 10 females with an average age of 58.2 ± 8.1 years (35–80 years) and an average lesion size of 3.1 ± 1.3 cm (1.0–5.0 cm). There were 38 patients with solitary lesions, and four had multiple lesions. The largest tumor was used to evaluate the CEUS features. The etiologies of liver disease included hepatitis B in 34 patients, hepatitis C in 4, alcoholic liver disease in 2, and absence of liver disease in 2. Of them, 31 (73.8%) patients had abnormal serum ALT/AST levels, and 15 (35.7%) patients had elevated AFP levels before RFA treatment. Thirty (71.4%) patients had Child-Pugh class A and 12 (28.6%) had class B. During the follow-up, these recurrent tumors occurred 2–65 months after initial RFA. With the exception of the tumor size, there was no significant difference in other clinical characteristics between the initial HCC and recurrent HCC groups (Table 1).

**Table 1:** The basic characteristics of initial HCC and recurrent HCC patients.

**Varies**	**Initial HCC**	**Recurrent HCC**	***p*-value**
No. of patients	42	42	
Gender			1.0
Male	32 (76.2)	32 (76.2)	
Female	10 (23.8)	10 (23.8)	
**Age**			
Mean	58.2 ± 8.1	58.7 ± 7.9	0.875
≤ 60 years	25 (59.5)	23 (54.8)	0.659
>60 years	17 (40.5)	19 (45.2)	
Tumor size^*^			
Mean	3.1 ± 1.3	2.5 ± 1.0	0.012
≤ 3 cm	20 (47.6)	31 (73.8)	0.014
3.1–5 cm	22 (52.4)	11 (26.2)	
Etiology of liver disease			0.776
HBV	34 (81.0)	35 (83.3)	
HCV	4 (9.5)	4 (9.5)	
Alcoholic liver	2 (4.8)	2 (4.8)	
Absence of liver disease	2 (4.8)	1 (2.4)	
Liver cirrhosis			0.763
None	7 (16.7)	6 (14.3)	
Yes	35 (83.3)	36 (85.7)	
Serum ALT/AST			0.474
Normal	31 (73.8)	28 (66.7)	
Elevated	11 (26.2)	14 (33.3)	
Child–Pugh class			0.355
Child-push A	30 (71.4)	26 (61.9)	
Child-push B	12 (28.6)	16 (38.1)	
Serum AFP level			0.818
≤ 20 ng/ml	27 (64.3)	28 (66.7)	
>20 ng/ml	15 (35.7)	14 (33.3)	

*HCC, hepatocellular carcinoma; HBV, hepatitis B virus; HCV, hepatitis C virus; ALT, alanine aminotransferase; AST, aspartate transaminase; AFP, alpha-fetoprotein. Numbers in parentheses are percentages*.

* *Statistically significant*.

### Comparison of CEUS Perfusion Features

**Figure 1:**
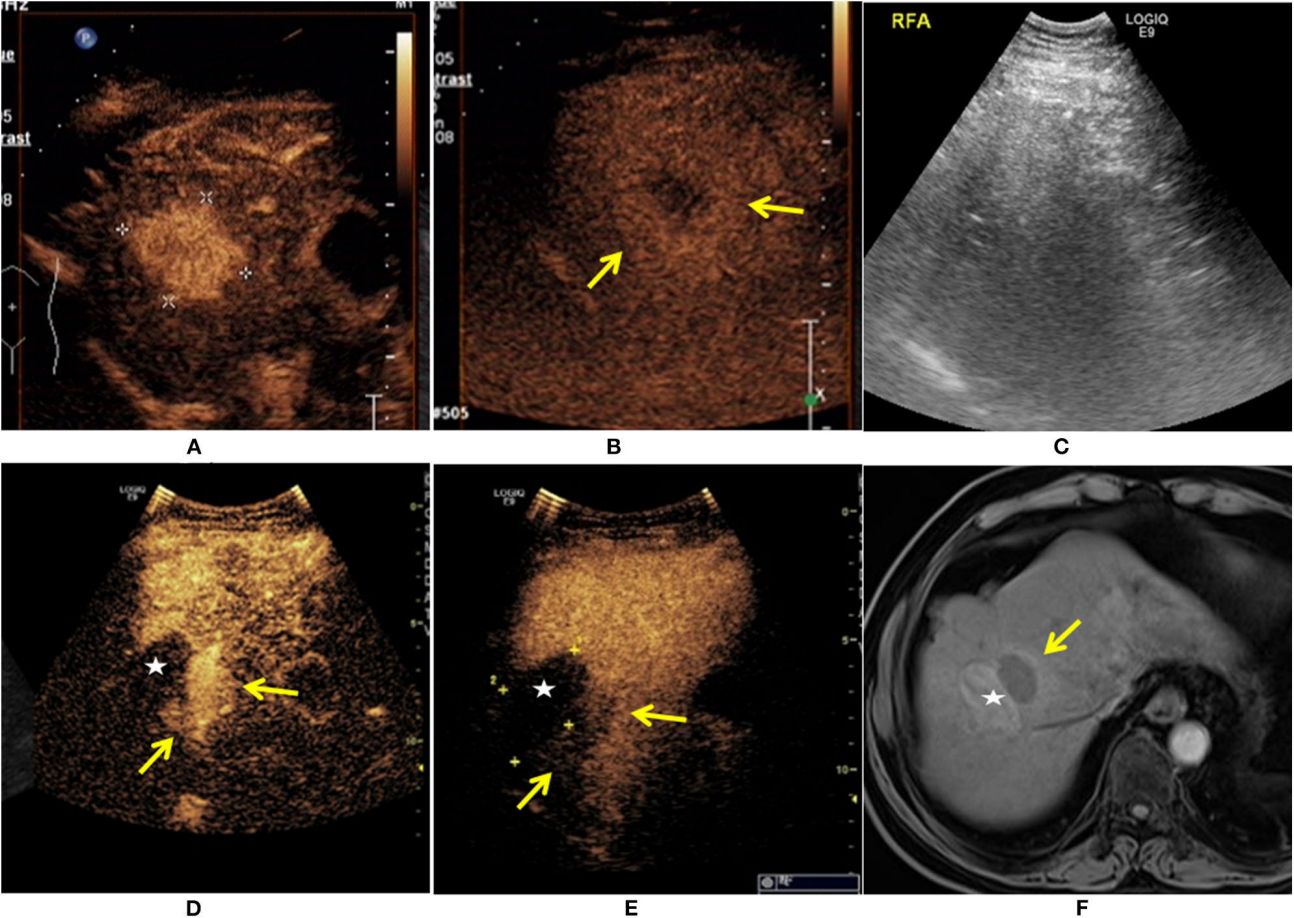
A 65-year-old man was diagnosed with HCC. The patient had a 30-year history of hepatitis B. The contrast-enhanced ultrasound (CEUS) showed a single lesion measuring 4.1 × 3.6 cm in size (+) enhanced at arterial phase **(A)**, and the mild washout in lesion was found at the late phase (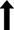) **(B)**. The patient received ultrasound-guided radiofrequency ablation (RFA) treatment **(C)**. Eleven months after RFA, there was an irregular enhancement (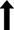) around the ablation zone (★), and marked washout at the late phase (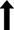) **(D,E)**. The local recurrence after RFA was also demonstrated at enhanced MRI (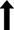) **(F)**.

**Figure 2:**
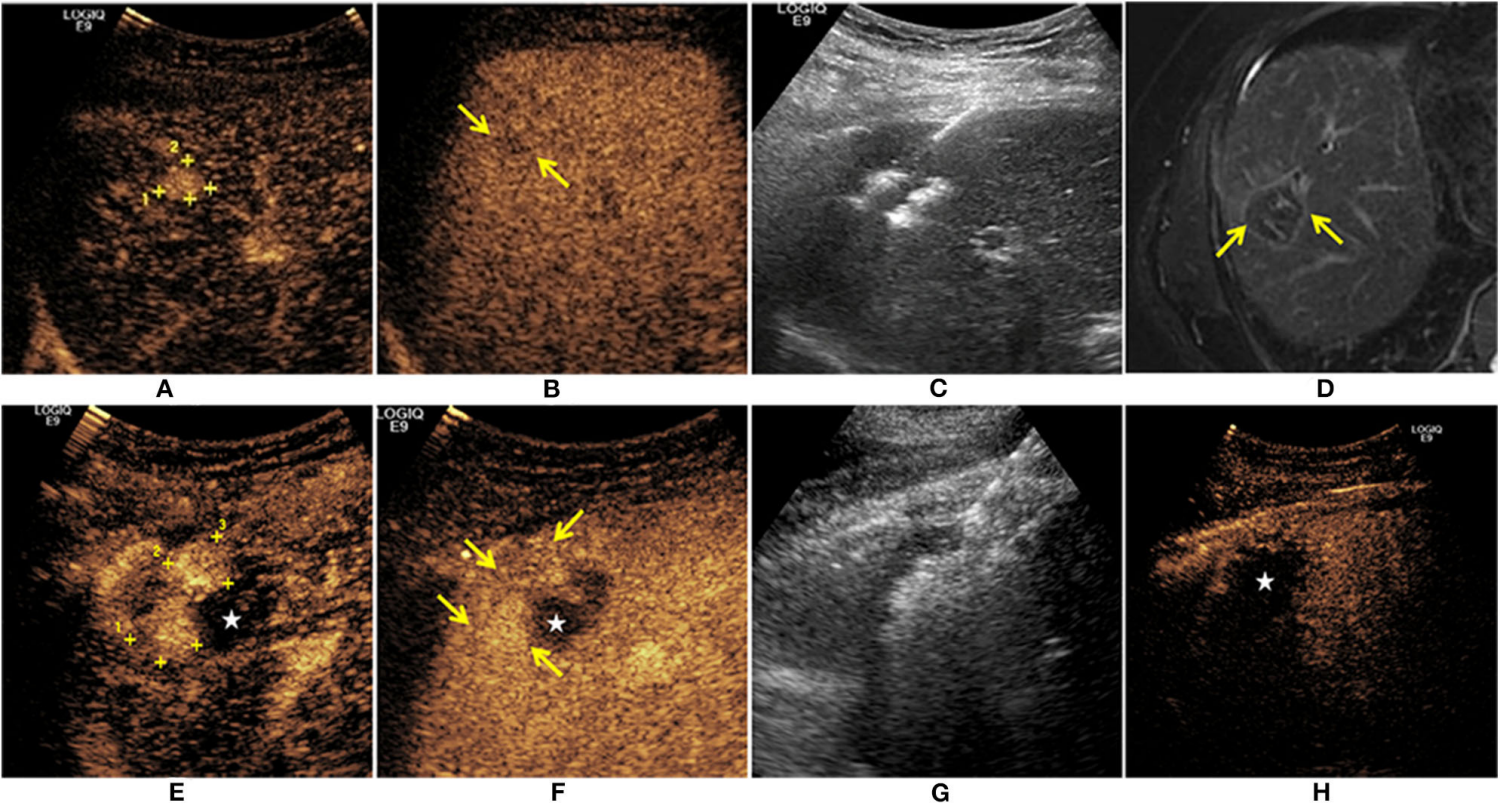
A 59-year-old man was diagnosed with HCC. The CEUS showed a single lesion (+) enhanced at arterial phase **(A)**, and the mild washout in the lesion was found at the late phase (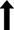) **(B)**. The patient received ultrasound-guided RFA treatment **(C)**. One month after treatment, there was no residual tumor on MR imaging (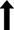) **(D)**. Thirteen months after RFA, there were irregular nodules enhanced (+) at the upper edge of the ablation zone (★) **(E)**. Mild washout was found at the late phase (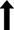) **(F)**. The second RFA for this recurrent HCC was performed under ultrasound guidance **(G)**. Immediate CEUS after RFA treatment showed that there was no enhancement in the ablation zone (★) **(H)**.

Of the 42 initial HCCs, all tumors had arterial phase enhancement, and 40 tumors had washout in the portal phase or late phase. On the other hand, all 42 recurrent HCC lesions showed arterial phase enhancement and washout in the portal phase or late phase. The comparison of CEUS findings is summarized in Table 2. In the arterial phase of CEUS, 59.5% of the initial HCC lesions showed centripetal enhancement, while 61.9% of the recurrent HCC lesions showed homogeneous enhancement (*p* < 0.001). At the peak, 73.8% of the initial HCC lesions had well-circumscribed margins, while 81.0% of the recurrent HCC lesions had poorly defined margins (*p* < 0.001). A total of 78.6% of the initial HCC lesions had regular morphology at the peak, while 83.3% of the recurrent HCC lesions were irregular (*p* < 0.001). Feeding vessels were more frequently visualized in initial HCC lesions (71.4%) than in recurrent HCC lesions (38.1%, *p* = 0.002). In the portal phase or late phase, 60% of initial HCCs had marked washout while 83.3% of recurrent HCCs had marked washout (*p* = 0.019). Thirty-one percent of the initial HCCs had internal necrosis areas, while only 7.1% of recurrent HCCs had internal necrosis areas (*p* = 0.035) (Figures 1, 2).

**Table 2:** The comparison of the perfusion features of CEUS between initial and recurrent HCC tumors.

**Perfusion features**		**Initial HCC**	**Recurrent HCC**	***p*-value**
**Arterial phase**				
**Enhancement**		42 (100)	42 (100)	1.0
Enhance process^*^				<0.001
	Centrifugal	4 (9.5)	4 (9.5)	
	Centripetal	25 (59.5)	6 (14.3)	
	Homogeneous	8 (19.0)	26 (61.9)	
	Others	5 (11.9)	6 (14.3)	
Morphology at peak^*^				<0.001
	Regular	33 (78.6)	7 (16.7)	
	Irregular	9 (21.4)	35 (83.3)	
Margin at peak^*^				<0.001
	Well defined	31 (73.8)	8 (19.0)	
	Poor defined	11 (26.2)	34 (81.0)	
Feeding vessels^*^				0.002
	Yes	30 (71.4)	16 (38.1)	
	No	12 (28.6)	26 (61.9)	
**PV or late phase**				
**Washout**		40^#^ (95.2)	42 (100)	0.494
Degree of washout^*^	Marked	24 (60.0)	35 (83.3)	0.019
	Mild	16 (40.0)	7 (16.7)	
Inner necrosis area^*^				0.035
	Yes	10 (31.3)	3 (7.1)	
	No	32 (68.7)	39 (92.9)	

*CEUS, contrast-enhanced ultrasound; HCC, hepatocellular carcinoma; PV, portal vein. Numbers in parentheses are percentages*.

* *Statistically significant*.

# *Two initial HCCs showed no marked or mild washout in the PV or late phase*.

### Comparison of CEUS Quantitative Parameters

Quantitative analysis of the TIC showed that there was no significant difference between initial HCC and recurrent HCC lesions in terms of enhancement time (16.5 ± 3.3 s vs. 17.4 ± 3.1 s, *p* = 0.256), peak time (23.7 ± 4.6 s vs. 24.4 ± 5.0 s, *p* = 0.384), and washout time (74.7 ± 38.6 s vs. 58.4 ± 18.5 s, *p* = 0.186). We further divided cases into two subgroups based on tumor size. In <3 cm tumors, there was no significant difference in TIC parameters between initial HCC and recurrent HCC lesions (*p* = 0.084, *p* = 0.913, and *p* = 0.076). In tumors 3–5 cm in size, the washout time in recurrent HCCs was shorter than that in initial HCCs (50.3 ± 13.5 s vs. 75.6 ± 45.8 s, *p* = 0.013) (Table 3).

**Table 3:** The comparison of the quantitative parameters of TIC between initial HCC and recurrent HCC tumors.

**Group**	**Tumor size: 1.0–3.0 cm**				**Tumor size: 3.1–5.0 cm**			
	***n***	**ET (s)**	**PT (s)**	**WT (s)**	***n***	**ET (s)**	**PT (s)**	WT (s)^*^
Initial HCC	20	15.8 ± 4.1	23.2 ± 5.3	74.1 ± 39.7	22	17.4 ± 3.7	24.6 ± 5.8	75.6 ± 45.8
Recurrent HCC	31	18.1 ± 3.7	24.3 ± 4.2	63.8 ± 29.1	11	17.0 ± 4.0	24.5 ± 5.3	50.3 ± 13.5
*p* value	—	0.084	0.913	0.076	—	0.925	0.874	0.013

*TIC, time–intensity curve; HCC, hepatocelluar carcinoma; ET, enhance time; PT, peak time; WT, washout time*.

* *Statistically significant*.

### Pathological Results

**Figure 3:**
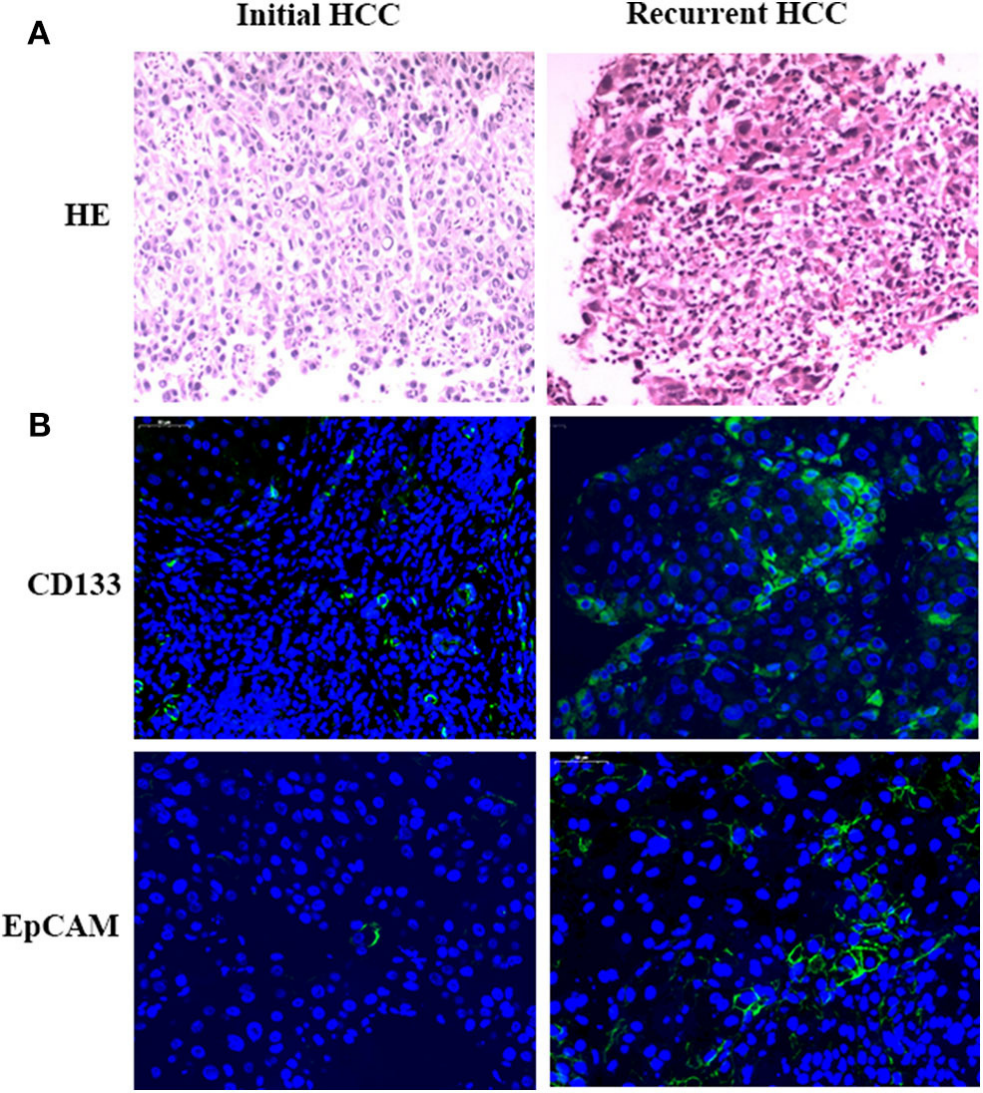
Pathological results. Tumor samples obtained from initial and recurrent HCC patients treated with RFA were examined by H&E staining. H&E staining showed the more intensive cell distribution detected in recurrent HCC compared with the initial tumor in the same patient **(A)**. Immunofluorescence staining was used for detecting the expression of CD133 and EpCAM in HCC tumor samples **(B)**. Positive cells were stained with green signal; nuclei were stained with DAPI (Blue). Magnification: 400×.

The HE staining results of HCC samples demonstrated that a more intensive cell distribution was detected in the recurrent HCC than in the initial tumor in the same patient (Figure 3A). Moreover, IF staining showed that the tumor stem cell markers CD133 and EpCAM were both highly expressed in recurrent samples compared with the expression in initial tumors (CD133+: 19 vs. 5%, *p* = 0.002; EpCAM+:15 vs. 6%, *p* = 0.005) (Figure 3B).

## Discussion

RFA, along with surgical resection and liver transplantation, has been recognized as one of the radical therapies for HCC, especially for small HCC. However, regardless of technical success, local tumor recurrence affects long-term survival after radical treatment (3, 21). In our center, the local recurrence rate of HCC after RFA in 10 years was 13.8% (21). The mechanisms of malignant behaviors from recurrent HCC after RFA have been increasingly reported (22). Early detection and diagnosis of local recurrence in HCC patients' follow-up have become important issues (5, 9, 23–26). A previous study (27) indicated that isoenhancement in all vascular phase patterns on CEUS was found in >50% of recurrent lesions, indicating a high risk of HCC. This suggests that recurrent lesions may be different from initial HCC lesion in terms of perfusion patterns. However, the comparison between initial HCC and recurrent HCC of CEUS performance has not been yet reported.

As previously reported, the performance of HCC is mainly fast-in and fast-out on CEUS and dynamically enhanced CT images (28, 29). Microvascular density in recurrent disease is significantly higher on CEUS evaluation than on CECT/MRI (*p* < 0.05) (9). Recurrent tumors have the characteristics of incipient lesions. Our study showed that all local recurrent lesions had different levels of enhancement, and all were consistent with the characteristics of malignant performance. However, the artery-portal phase enhancement and late washout pattern were not sufficient to assess the characteristics of recurrence after RFA treatment.

In our study, we analyzed in more detail the CEUS performance for recurrent HCC after RFA and compared it with that for initial HCC before treatment. The results showed the unique performance of the enhancement process, pattern, lesion border, internal necrosis, and feeding vessels. Reported animal studies have showed that thermal ablation promotes a large degree of blood sinus expansion surrounding the ablation area and results in microvascular structure disorder hyperplasia. Kong et al. reported (16) that angiogenesis produced by altered cells after hyperthermia treatment through the HIF1α/VEGFA pathway could be one of the vital factors causing the rapid growth of residual HCC after RFA. With the wide application of CEUS in the clinic, especially the application of microvascular flow imaging (30, 31), Kang et al. demonstrated (31) significantly higher sensitivity and accuracy than color Doppler imaging and power Doppler imaging for the detection of intratumoral vascularity in suspected residual or recurrent HCCs. Benefiting from our center's regular follow-up after RFA treatment, local tumor recurrence can be found in the earlier stage. Therefore, the tumor size in the recurrent HCC group was smaller than that in the initial HCC group. The difference in tumor size might partly be attributed to the different patterns of blood perfusion on CEUS imaging. The tumor was larger, and the frequency of the feeding artery was increased. The absence of feeding vessels between initial HCC and recurrent HCC was significantly different (*p* = 0.002). Additionally, 31.3% of the initial HCC lesions had internal necrosis areas, while only 7.1% of recurrent HCC had internal necrosis areas (*p* = 0.035). Accordingly, in the arterial phase, the enhancement process was different [initial HCC group: centripetal (59.5%), recurrent HCC group: homogeneous (61.9%), *p* < 0.001].

In addition to tumor size, incomplete RFA enhanced the invasiveness and metastasis of residual cancer in HCC cells (17, 32). The aggressive biological behavior of recurrent tumors might cause poorly defined margins and irregular lesions on CEUS. In the diagnostic algorithm for focal liver lesions on CEUS, washout was an important feature and was highly suggestive of malignancy. Kitao et al. (33) reported that during multistep hepatocarcinogenesis, the drainage vessels of HCC changed from hepatic veins to hepatic sinusoids, and then to portal veins in the course of dedifferentiation. They (33) suggested that the drainage vessels of HCC changed from hepatic veins to hepatic sinusoids, which may be correlated with the onset of tumor washout on CEUS. Accordingly, when the hepatic sinusoids became the main drainage vessels, late washout occurred. In advanced lesions, the portal veins became the main drainage vessels, and fast washout was frequently seen. Our study showed that the washout time in recurrent HCCs was shorter than that in initial HCCs with a tumor size of 3–5 cm (50.3 ± 13.5 s vs. 75.6 ± 45.8 s, *p* = 0.013) and the marked washout rate was higher in recurrent HCCs (83.3 vs. 60%, *p* = 0.019), which is consistent with previous studies. The clinical significance of our CEUS study on recurrent HCC was as follows: first, a summary of the perfusion features of recurrent HCC on CEUS would help to differentiate diagnosis between recurrent HCC and other diseases, such as inflammatory reactions. Second, investigating the difference in perfusion features of recurrent HCC compared with initial HCC would help to understand the biological behavior of recurrent HCC. Third, identifying the tumor range and feeding vessels also provided useful information to plan the treatment protocol for local therapy.

Additionally, we found that the tumor stem cell markers (CD133 and EpCAM) were both highly expressed in recurrent HCCs compared with initial HCCs (CD133+: 19 vs. 5%, *p* = 0.002; EpCAM+: 15 vs. 6%, *p* = 0.005). These results provide a reference for clinical practice. According to a previous study (26), the expression levels of basic fibroblast growth factor in recurrent HCC were significantly higher than those in non-recurrent HCC (*p* < 0.05) and were associated with HCC recurrence after RFA. Thus, the biological behavior of recurrent HCCs is more aggressive than that of primary HCCs. It would be more difficult to treat recurrent lesions with local ablation. Additionally, the safety ablation margins should have been larger when we performed the second RFA session.

There were some limitations in our study. First, the study was retrospective with some inevitable bias due to a single center. Second, recurrent tumors in difficult locations might have been missed by CEUS because of the inherent limitation of CEUS examination. In addition, due to the small sample size, we did not further analyze the degree of HCC differentiation or the local recurrence time (early recurrence vs. late recurrence). The local recurrence rate after RFA in our center was low. More patient data need to be collected in further studies.

In conclusion, recurrent HCC after RFA had more homogeneous enhancement, a poorly defined border, marked washout, and fewer feeding vessels and inner necrosis areas. These specific perfusion features might reflect the aggressive biological behavior and higher expression of cancer stem cell markers in recurrent HCC. Additionally, the analysis of CEUS findings for local recurrence after RFA would be useful for the differential diagnosis between inflammatory reaction and tumor recurrence as well as for identifying recurrence early. Furthermore, the perfusion feature in local recurrence of HCC would help to design optimal treatment strategies.

## Data Availability Statement

All datasets presented in this study are included in the article/supplementary material.

## Ethics Statement

The studies involving human participants were reviewed and approved by the institutional review board of Peking University Cancer Hospital. The patients/participants provided their written informed consent to participate in this study.

## Author Contributions

WW and KY performed the patient's CEUS examinations. J-YW, X-MB, and WY helped to design the study and reviewed the images and performed the statistical analysis. HW and SW organized and analyzed the imaging database. J-YW, X-MB, and QX wrote the first draft of the manuscript. All authors contributed to the article and approved the submitted version.

## Conflict of Interest

The authors declare that the research was conducted in the absence of any commercial or financial relationships that could be construed as a potential conflict of interest.
